# Small-Pox in Atlanta

**Published:** 1894-04

**Authors:** 


### SMALL-POX IN ATLANTA.

On the first of March one case of small-pox was discovered in the western part of the city. The origin of the infection it was not possible to trace with certainty. The Board of Health immediately instituted the most active measures to limit and control the disease. All persons who had been exposed to the first patient were at once vaccinated, and the houses containing such persons were put under quarantine. In spite of such measures other cases developed in the neighborhood and in other parts of the city. A vaccinating corps was organized, consisting of about twenty-five physicians, who were instructed to lose no time in getting the city thoroughly vaccinated. These gentlemen proceeded with their unpleasant task in earnest, and in three weeks had vaccinated about twenty-two thousand persons. Their work was made very much more difficult from the fact that the daily press of the city carefully kept the people in ignorance as to the existence of the disease among us. It was a remark heard many times a day by those doing house-to-house vaccination, "Don't believe there is any small-pox here; the papers don't say anything about it." The papers declined even to publish the daily reports from the Board of Health. In the meantime outside the city the most exaggerated reports were current concerning the prevalence of the disease in Atlanta.

All told, there have been twenty-two cases in the city, this number embracing all cases of *variola vera* and *varioloid*. With the exception of the first two or three cases the subsequent patients were transferred to the city pest-house as soon as discovered. Here they have been under the constant attention of a physician detailed by the Board of Health. All patients have received the best of both medical treatment and nursing, and there have been no deaths.

In this connection we may mention that the law of the city requiring all children to be successfully vaccinated before they can enter the public schools is in a great measure nugatory. It is customary with the physician vaccinating children to give a certificate of successful vaccination as soon as he has finished with his little operation. The children enter school, and often the vaccination doesn't "take." The writer and his associates went carefully through the public schools of one ward of the city, and we estimate that at least thirty per cent. of the children showed no indication of successful vaccination. Doubtless this estimate is correct as applied to all the public schools of the city. Other cities and towns probably have the same law nominally in force, but with the same defect in operation. The remedy is to be found in the physician's withholding the certificate until he is sure of a successful vaccination.

